# 3-(2-Meth­oxy­phen­yl)-2,3-di­hydro-1*H*-benzo[*f*]chromen-1-one

**DOI:** 10.1107/S2414314620012092

**Published:** 2020-09-08

**Authors:** Jiha Sung

**Affiliations:** a Department of Applied Chemistry, Dongduk Women’s University, Seoul 136-714, Republic of Korea; University of Aberdeen, Scotland

**Keywords:** crystal structure, flavanone, C—H⋯O hydrogen bonds

## Abstract

In the title compound, C_20_H_16_O_3_, the 2-meth­oxy­phenyl ring is tilted by 50.67 (3)° with respect to the naphthyl ring system. The central pyran ring has an envelope conformation with the C atom bearing the pendant ring system as the flap. The meth­oxy group attached to the benzene ring is slightly twisted [C—C—O—C = −15.2 (1)°] from the ring. In the crystal, weak C—H**⋯**O inter­actions link the mol­ecules into *C*(7) chains propagating along [101].

## Abstract 2

In the title flavanone, the mol­ecules are linked by weak C—H⋯O inter­actions into [101] chains.

## Graphical abstract

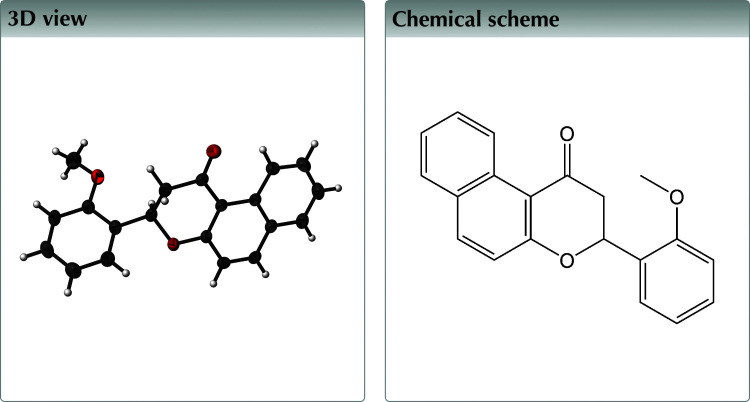

## Structure description

Flavanones are widely used as health-care products because they are found at high concentrations in natural sources (Lichota *et al.*, 2019 ▸). Flavanones possess a chromane ring as a common structural feature, but they show a broad spectrum of biological activities depending on the placement of the hydroxyl or meth­oxy group substituents at different positions of the flavanone skeleton (Lee *et al.*, 2016 ▸; Singh *et al.*, 2014 ▸). Compounds in which the phenyl group in the chromane ring system is replaced by a naphthyl ring system have shown versatile biological activities and physiochemical properties (Kumar *et al.*, 2017 ▸; Shin *et al.*, 2014 ▸). Therefore, the naphthyl ring system-containing title flavanone compound, C_20_H_16_O_3_, was synthesized and its crystal structure was determined.

**Figure 1:**
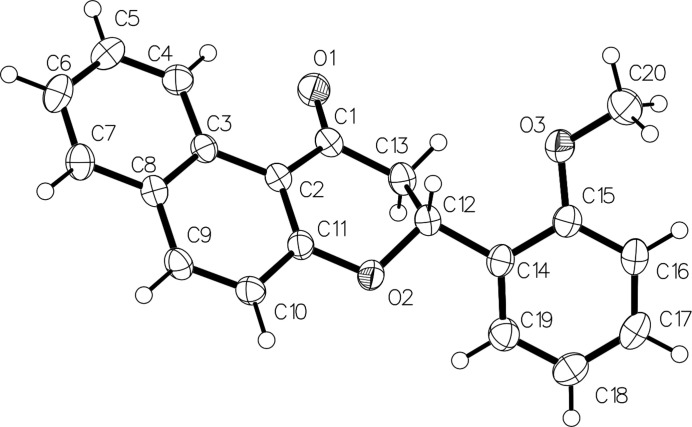
The mol­ecular structure of the title compound with displacement ellipsoids drawn at the 30% probability level.

The mol­ecular structure of the title compound is shown in Fig. 1 ▸. The dihedral angle between the C2–C11 naphthyl ring system (r.m.s. deviation = 0.026 Å) and the C14–C19 2-meth­oxy­phenyl ring is 50.67 (3)°. The central pyran ring (C1/C2/C11/O2/C12/C13) has an envelope conformation with atom C12 as the flap, which is displaced by 0.691 (2) Å from the mean plane of the other five atoms (r.m.s. deviation = 0.023 Å). In the arbitrarily chosen asymmetric mol­ecule, C12 has an *R* configuration but crystal symmetry generates a racemic mixture. The hydrogen atom H12 attached to C12 forms a *trans* diaxial conformation with one of H atoms of the C13 methyl­ene group (H12—C12—C13—H13*A* = 179°] and a *gauche* conformation with the other methyl­ene H atom H13*B* (H12—C12—C13—H13*B* = 61°). The meth­oxy group in the benzene ring is slightly tilted [C16—C15—O3—C20 = −15.2 (2)°] from the ring.

**Figure 2:**
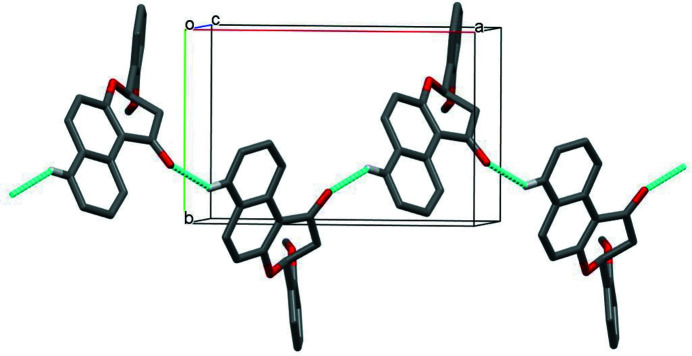
Part of the crystal structure of the title compound, showing the weak C—H⋯O hydrogen bonds as blue lines. H atoms not involved in these inter­actions have been omitted for clarity.

In the crystal, weak C—H⋯O inter­actions link the mol­ecules into *C*(7) chains propagating along [101] (Table 1 ▸, Fig. 2 ▸) with adjacent mol­ecules in the chain related by *n*-glide symmetry.

## Synthesis and crystallization

**Figure 3:**
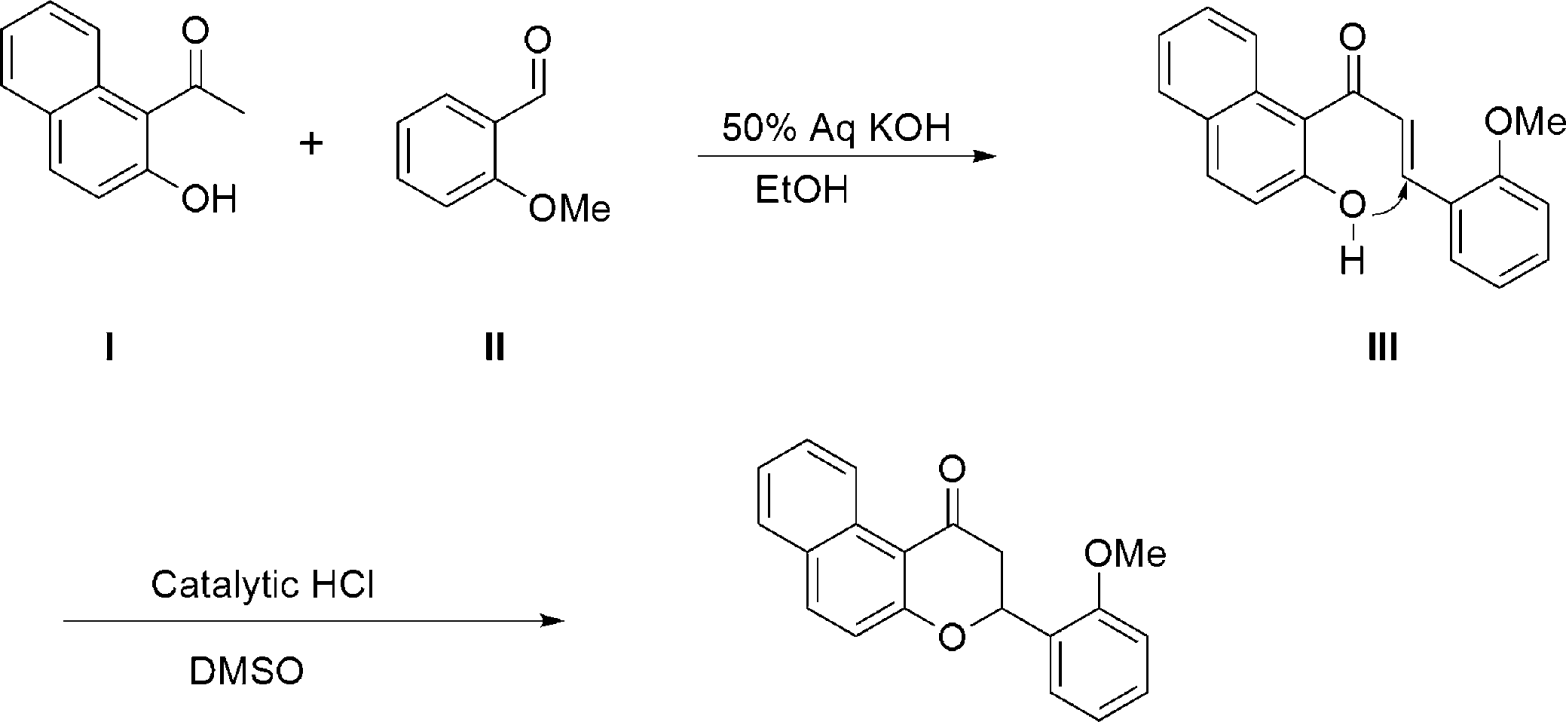
A synthetic scheme for the preparation of the title compound.

The synthetic scheme for the preparation of the title compound is shown in Fig. 3 ▸: 2-hy­droxy-1-aceto­naphthone (**I**, 372 mg, 2 mmol) and 2-meth­oxy­benzaldehyde (**II**, 272 mg, 2 mmol) were dissolved in ethanol (20 ml) and the temperature was adjusted to around 276–277 K in an ice-bath. To the cooled reaction mixture was added 1.5 ml of 50% aqueous KOH solution, and the reaction mixture was stirred at room temperature for 24 h. The mixture was poured into iced water (80 ml) and was acidified with 6 *N* HCl solution. The mixture was extracted with ethyl acetate (3 × 40 ml) and the combined organic layers were dried with MgSO_4_. Filtration and evaporation of the filtrate gave a solid product of chalcone (**III**), which was used for next reaction: the solid was dissolved in DMSO and a catalytic amount of conc. HCl was added. After stirring for 10 h, the reaction mixture was poured into iced water to give a solid product of the title flavanone and yellow blocks were recovered by recrystallization from ethanol solution.

## Refinement

Crystal data, data collection and structure refinement details are summarized in Table 2 ▸.

## Supplementary Material

Crystal structure: contains datablock(s) I. DOI: 10.1107/S2414314620012092/hb4362sup1.cif

Structure factors: contains datablock(s) I. DOI: 10.1107/S2414314620012092/hb4362Isup2.hkl

Supporting information file. DOI: 10.1107/S2414314620012092/hb4362Isup3.cml

CCDC reference: 2026731

Additional supporting information:  crystallographic information; 3D view; checkCIF report

## Figures and Tables

**Table 1:** Hydrogen-bond geometry (Å, °)

*D*—H⋯*A*	*D*—H	H⋯*A*	*D*⋯*A*	*D*—H⋯*A*
C7—H7⋯O1^i^	0.94	2.60	3.3973 (17)	142

Symmetry code: (i) 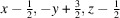 .

**Table 2:** Experimental details

Crystal data	
Chemical formula	C_20_H_16_O_3_
*M*_r_	304.33
Crystal system, space group	Monoclinic, *P*2_1_/*n*
Temperature (K)	223
*a*, *b*, *c* (Å)	12.4519 (5), 7.8785 (3), 15.6680 (7)
β (°)	105.2534 (16)
*V* (Å^3^)	1482.92 (11)
*Z*	4
Radiation type	Mo *K*α
μ (mm^−1^)	0.09
Crystal size (mm)	0.21 × 0.14 × 0.10

Data collection	
Diffractometer	PHOTON 100 CMOS
Absorption correction	Multi-scan (*SADABS*; Bruker, 2012 ▸)
*T*_min_, *T*_max_	0.691, 0.746
No. of measured, independent and observed [*I* > 2σ(*I*)] reflections	40884, 3704, 2879
*R*_int_	0.049
(sin θ/λ)_max_ (Å^−1^)	0.668

Refinement	
*R*[*F* ^2^ > 2σ(*F* ^2^)], *wR*(*F* ^2^), *S*	0.042, 0.113, 1.03
No. of reflections	3704
No. of parameters	209
H-atom treatment	H-atom parameters constrained
Δρ_max_, Δρ_min_ (e Å^−3^)	0.32, −0.20

Computer programs: *APEX2* and *SAINT* (Bruker, 2012 ▸), *SHELXS* and *SHELXTL* (Sheldrick, 2008 ▸), *SHELXL2014* (Sheldrick, 2015 ▸)and *publCIF* (Westrip, 2010 ▸).

## full crystallographic data

### Crystal data

**Table:**

C_20_H_16_O_3_	*F*(000) = 640
*M**_r_* = 304.33	*D*_x_ = 1.363 Mg m^−^^3^
Monoclinic, *P*2_1_/*n*	Mo *K*α radiation, λ = 0.71073 Å
*a* = 12.4519 (5) Å	Cell parameters from 9975 reflections
*b* = 7.8785 (3) Å	θ = 2.7–28.3°
*c* = 15.6680 (7) Å	µ = 0.09 mm^−^^1^
β = 105.2534 (16)°	*T* = 223 K
*V* = 1482.92 (11) Å^3^	Block, yellow
*Z* = 4	0.21 × 0.14 × 0.10 mm

### Data collection

**Table:**

PHOTON 100 CMOS diffractometer	2879 reflections with *I* > 2σ(*I*)
φ and ω scans	*R*_int_ = 0.049
Absorption correction: multi-scan (SADABS; Bruker, 2012)	θ_max_ = 28.4°, θ_min_ = 2.7°
*T*_min_ = 0.691, *T*_max_ = 0.746	*h* = −16→16
40884 measured reflections	*k* = −10→10
3704 independent reflections	*l* = −20→20

### Refinement

**Table:**

Refinement on *F*^2^	Primary atom site location: structure-invariant direct methods
Least-squares matrix: full	Hydrogen site location: inferred from neighbouring sites
*R*[*F*^2^ > 2σ(*F*^2^)] = 0.042	H-atom parameters constrained
*wR*(*F*^2^) = 0.113	*w* = 1/[σ^2^(*F*_o_^2^) + (0.0466*P*)^2^ + 0.6269*P*] where *P* = (*F*_o_^2^ + 2*F*_c_^2^)/3
*S* = 1.02	(Δ/σ)_max_ < 0.001
3704 reflections	Δρ_max_ = 0.32 e Å^−^^3^
209 parameters	Δρ_min_ = −0.20 e Å^−^^3^
0 restraints	

### Special details

**Table:**

Geometry. All esds (except the esd in the dihedral angle between two l.s. planes) are estimated using the full covariance matrix. The cell esds are taken into account individually in the estimation of esds in distances, angles and torsion angles; correlations between esds in cell parameters are only used when they are defined by crystal symmetry. An approximate (isotropic) treatment of cell esds is used for estimating esds involving l.s. planes.

### Fractional atomic coordinates and isotropic or equivalent isotropic displacement parameters (Å^2^)

**Table:**

	*x*	*y*	*z*	*U*_iso_*/*U*_eq_	
O1	1.02968 (9)	0.67451 (14)	0.13018 (7)	0.0408 (3)	
C1	0.96744 (11)	0.55251 (17)	0.11882 (8)	0.0277 (3)	
C2	0.87200 (10)	0.52818 (17)	0.04044 (8)	0.0254 (3)	
C3	0.83775 (10)	0.65224 (17)	−0.02963 (8)	0.0256 (3)	
C4	0.88498 (11)	0.81647 (18)	−0.02699 (9)	0.0319 (3)	
H4	0.9442	0.8475	0.0214	0.038*	
C5	0.84549 (12)	0.93097 (19)	−0.09406 (10)	0.0373 (3)	
H5	0.8778	1.0394	−0.0906	0.045*	
C6	0.75818 (12)	0.8889 (2)	−0.16720 (10)	0.0383 (3)	
H6	0.7318	0.9687	−0.2125	0.046*	
C7	0.71140 (12)	0.7314 (2)	−0.17257 (9)	0.0344 (3)	
H7	0.6531	0.7029	−0.2221	0.041*	
C8	0.74920 (11)	0.61053 (18)	−0.10475 (8)	0.0279 (3)	
C9	0.69968 (11)	0.44738 (19)	−0.11078 (9)	0.0312 (3)	
H9	0.6434	0.4186	−0.1616	0.037*	
C10	0.73167 (11)	0.33232 (18)	−0.04500 (9)	0.0308 (3)	
H10	0.6983	0.2245	−0.0504	0.037*	
C11	0.81568 (11)	0.37503 (17)	0.03210 (8)	0.0268 (3)	
O2	0.83415 (8)	0.25362 (12)	0.09597 (6)	0.0327 (2)	
C12	0.88068 (11)	0.31806 (18)	0.18418 (8)	0.0284 (3)	
H12	0.8275	0.4002	0.1981	0.034*	
C13	0.98756 (11)	0.41041 (18)	0.18597 (9)	0.0301 (3)	
H13A	1.0414	0.3302	0.1732	0.036*	
H13B	1.0193	0.4572	0.2452	0.036*	
C14	0.89365 (11)	0.17138 (18)	0.24762 (9)	0.0290 (3)	
C15	0.89304 (11)	0.20426 (18)	0.33543 (9)	0.0301 (3)	
C16	0.90122 (12)	0.0723 (2)	0.39537 (10)	0.0352 (3)	
H16	0.9000	0.0947	0.4540	0.042*	
C17	0.91114 (12)	−0.0927 (2)	0.36806 (10)	0.0381 (3)	
H17	0.9166	−0.1824	0.4086	0.046*	
C18	0.91317 (13)	−0.1273 (2)	0.28268 (11)	0.0398 (3)	
H18	0.9203	−0.2397	0.2649	0.048*	
C19	0.90462 (12)	0.00531 (19)	0.22271 (10)	0.0358 (3)	
H19	0.9063	−0.0184	0.1643	0.043*	
O3	0.88502 (9)	0.37221 (13)	0.35620 (7)	0.0398 (3)	
C20	0.85783 (17)	0.4093 (2)	0.43593 (11)	0.0520 (5)	
H20A	0.7929	0.3441	0.4391	0.078*	
H20B	0.8418	0.5295	0.4381	0.078*	
H20C	0.9200	0.3799	0.4855	0.078*	

### Atomic displacement parameters (Å^2^)

**Table:**

	*U*^11^	*U*^22^	*U*^33^	*U*^12^	*U*^13^	*U*^23^
O1	0.0417 (6)	0.0414 (6)	0.0332 (6)	−0.0146 (5)	−0.0011 (4)	0.0012 (5)
C1	0.0279 (6)	0.0315 (7)	0.0237 (6)	−0.0012 (5)	0.0070 (5)	−0.0024 (5)
C2	0.0248 (6)	0.0290 (7)	0.0224 (6)	−0.0004 (5)	0.0063 (5)	−0.0012 (5)
C3	0.0255 (6)	0.0289 (7)	0.0237 (6)	0.0009 (5)	0.0086 (5)	−0.0009 (5)
C4	0.0314 (7)	0.0320 (7)	0.0317 (7)	−0.0033 (6)	0.0072 (5)	−0.0011 (6)
C5	0.0384 (8)	0.0297 (7)	0.0447 (8)	−0.0017 (6)	0.0124 (6)	0.0056 (6)
C6	0.0364 (8)	0.0393 (8)	0.0391 (8)	0.0066 (6)	0.0094 (6)	0.0141 (7)
C7	0.0299 (7)	0.0422 (8)	0.0289 (7)	0.0034 (6)	0.0036 (5)	0.0060 (6)
C8	0.0258 (6)	0.0338 (7)	0.0242 (6)	0.0011 (5)	0.0066 (5)	0.0008 (5)
C9	0.0289 (7)	0.0384 (8)	0.0235 (6)	−0.0041 (6)	0.0022 (5)	−0.0024 (6)
C10	0.0317 (7)	0.0315 (7)	0.0280 (7)	−0.0075 (5)	0.0059 (5)	−0.0031 (6)
C11	0.0287 (6)	0.0288 (6)	0.0233 (6)	0.0001 (5)	0.0078 (5)	0.0012 (5)
O2	0.0403 (5)	0.0299 (5)	0.0248 (5)	−0.0056 (4)	0.0033 (4)	0.0037 (4)
C12	0.0297 (6)	0.0319 (7)	0.0228 (6)	0.0005 (5)	0.0054 (5)	0.0019 (5)
C13	0.0273 (6)	0.0369 (7)	0.0248 (6)	0.0000 (6)	0.0044 (5)	0.0019 (5)
C14	0.0257 (6)	0.0322 (7)	0.0286 (7)	0.0021 (5)	0.0064 (5)	0.0051 (6)
C15	0.0274 (6)	0.0326 (7)	0.0305 (7)	0.0004 (5)	0.0083 (5)	0.0036 (6)
C16	0.0352 (7)	0.0413 (8)	0.0285 (7)	−0.0005 (6)	0.0073 (6)	0.0078 (6)
C17	0.0354 (7)	0.0359 (8)	0.0419 (8)	0.0025 (6)	0.0083 (6)	0.0143 (7)
C18	0.0409 (8)	0.0316 (7)	0.0465 (9)	0.0056 (6)	0.0110 (7)	0.0039 (7)
C19	0.0380 (8)	0.0365 (8)	0.0329 (7)	0.0045 (6)	0.0095 (6)	0.0019 (6)
O3	0.0562 (7)	0.0342 (6)	0.0333 (6)	0.0010 (5)	0.0192 (5)	0.0029 (4)
C20	0.0748 (12)	0.0454 (10)	0.0433 (9)	0.0065 (9)	0.0290 (9)	−0.0019 (8)

### Geometric parameters (Å, º)

**Table:**

O1—C1	1.2180 (16)	O2—C12	1.4430 (16)
C1—C2	1.4796 (17)	C12—C14	1.5050 (18)
C1—C13	1.5114 (18)	C12—C13	1.5105 (18)
C2—C11	1.3844 (18)	C12—H12	0.9900
C2—C3	1.4478 (18)	C13—H13A	0.9800
C3—C4	1.4173 (19)	C13—H13B	0.9800
C3—C8	1.4235 (18)	C14—C19	1.382 (2)
C4—C5	1.374 (2)	C14—C15	1.4020 (19)
C4—H4	0.9400	C15—O3	1.3725 (17)
C5—C6	1.396 (2)	C15—C16	1.3869 (19)
C5—H5	0.9400	C16—C17	1.384 (2)
C6—C7	1.364 (2)	C16—H16	0.9400
C6—H6	0.9400	C17—C18	1.372 (2)
C7—C8	1.4123 (19)	C17—H17	0.9400
C7—H7	0.9400	C18—C19	1.390 (2)
C8—C9	1.4179 (19)	C18—H18	0.9400
C9—C10	1.3515 (19)	C19—H19	0.9400
C9—H9	0.9400	O3—C20	1.4083 (18)
C10—C11	1.4149 (18)	C20—H20A	0.9700
C10—H10	0.9400	C20—H20B	0.9700
C11—O2	1.3595 (15)	C20—H20C	0.9700
			
O1—C1—C2	124.39 (12)	C14—C12—C13	114.63 (11)
O1—C1—C13	120.01 (12)	O2—C12—H12	108.5
C2—C1—C13	115.57 (11)	C14—C12—H12	108.5
C11—C2—C3	118.42 (11)	C13—C12—H12	108.5
C11—C2—C1	117.88 (12)	C12—C13—C1	111.10 (11)
C3—C2—C1	123.64 (12)	C12—C13—H13A	109.4
C4—C3—C8	117.39 (12)	C1—C13—H13A	109.4
C4—C3—C2	123.84 (12)	C12—C13—H13B	109.4
C8—C3—C2	118.73 (12)	C1—C13—H13B	109.4
C5—C4—C3	120.98 (13)	H13A—C13—H13B	108.0
C5—C4—H4	119.5	C19—C14—C15	118.58 (13)
C3—C4—H4	119.5	C19—C14—C12	122.80 (12)
C4—C5—C6	121.10 (14)	C15—C14—C12	118.61 (12)
C4—C5—H5	119.4	O3—C15—C16	124.01 (13)
C6—C5—H5	119.4	O3—C15—C14	115.49 (12)
C7—C6—C5	119.62 (14)	C16—C15—C14	120.50 (13)
C7—C6—H6	120.2	C17—C16—C15	119.44 (14)
C5—C6—H6	120.2	C17—C16—H16	120.3
C6—C7—C8	120.98 (13)	C15—C16—H16	120.3
C6—C7—H7	119.5	C18—C17—C16	120.90 (14)
C8—C7—H7	119.5	C18—C17—H17	119.5
C7—C8—C9	120.54 (12)	C16—C17—H17	119.5
C7—C8—C3	119.91 (13)	C17—C18—C19	119.50 (15)
C9—C8—C3	119.54 (12)	C17—C18—H18	120.2
C10—C9—C8	121.43 (12)	C19—C18—H18	120.2
C10—C9—H9	119.3	C14—C19—C18	121.06 (14)
C8—C9—H9	119.3	C14—C19—H19	119.5
C9—C10—C11	119.67 (13)	C18—C19—H19	119.5
C9—C10—H10	120.2	C15—O3—C20	117.37 (12)
C11—C10—H10	120.2	O3—C20—H20A	109.5
O2—C11—C2	124.08 (12)	O3—C20—H20B	109.5
O2—C11—C10	113.89 (12)	H20A—C20—H20B	109.5
C2—C11—C10	122.02 (12)	O3—C20—H20C	109.5
C11—O2—C12	113.89 (10)	H20A—C20—H20C	109.5
O2—C12—C14	107.98 (11)	H20B—C20—H20C	109.5
O2—C12—C13	108.49 (10)		
			
O1—C1—C2—C11	173.33 (13)	C9—C10—C11—C2	−4.2 (2)
C13—C1—C2—C11	−4.76 (17)	C2—C11—O2—C12	24.36 (17)
O1—C1—C2—C3	−3.7 (2)	C10—C11—O2—C12	−155.02 (11)
C13—C1—C2—C3	178.17 (11)	C11—O2—C12—C14	178.52 (10)
C11—C2—C3—C4	176.81 (12)	C11—O2—C12—C13	−56.70 (14)
C1—C2—C3—C4	−6.14 (19)	O2—C12—C13—C1	57.80 (14)
C11—C2—C3—C8	−1.15 (18)	C14—C12—C13—C1	178.55 (11)
C1—C2—C3—C8	175.91 (12)	O1—C1—C13—C12	154.04 (13)
C8—C3—C4—C5	0.76 (19)	C2—C1—C13—C12	−27.78 (16)
C2—C3—C4—C5	−177.22 (13)	O2—C12—C14—C19	24.76 (17)
C3—C4—C5—C6	−0.4 (2)	C13—C12—C14—C19	−96.26 (16)
C4—C5—C6—C7	−0.3 (2)	O2—C12—C14—C15	−154.33 (12)
C5—C6—C7—C8	0.6 (2)	C13—C12—C14—C15	84.64 (15)
C6—C7—C8—C9	179.72 (14)	C19—C14—C15—O3	178.25 (12)
C6—C7—C8—C3	−0.3 (2)	C12—C14—C15—O3	−2.62 (18)
C4—C3—C8—C7	−0.40 (18)	C19—C14—C15—C16	−1.2 (2)
C2—C3—C8—C7	177.69 (12)	C12—C14—C15—C16	177.95 (12)
C4—C3—C8—C9	179.58 (12)	O3—C15—C16—C17	−178.74 (13)
C2—C3—C8—C9	−2.33 (18)	C14—C15—C16—C17	0.6 (2)
C7—C8—C9—C10	−177.34 (13)	C15—C16—C17—C18	0.1 (2)
C3—C8—C9—C10	2.7 (2)	C16—C17—C18—C19	−0.3 (2)
C8—C9—C10—C11	0.5 (2)	C15—C14—C19—C18	1.0 (2)
C3—C2—C11—O2	−174.87 (11)	C12—C14—C19—C18	−178.10 (13)
C1—C2—C11—O2	7.90 (19)	C17—C18—C19—C14	−0.3 (2)
C3—C2—C11—C10	4.46 (19)	C16—C15—O3—C20	−15.2 (2)
C1—C2—C11—C10	−172.76 (12)	C14—C15—O3—C20	165.38 (14)
C9—C10—C11—O2	175.16 (12)		

### Hydrogen-bond geometry (Å, º)

**Table:**

*D*—H···*A*	*D*—H	H···*A*	*D*···*A*	*D*—H···*A*
C7—H7···O1^i^	0.94	2.60	3.3973 (17)	142

Symmetry code: (i) *x*−1/2, −*y*+3/2, *z*−1/2.
